# Coarse Grain Consumption and Risk of Cardiometabolic Diseases: A Prospective Cohort Study of Chinese Adults

**DOI:** 10.1093/jn/nxac041

**Published:** 2022-03-02

**Authors:** Jiaomei Yang, Huaidong Du, Yu Guo, Zheng Bian, Canqing Yu, Yiping Chen, Ling Yang, Jiben Liu, Xianyong Han, Junshi Chen, Jun Lv, Liming Li, Zhengming Chen, for the China Kadoorie Biobank Collaborative Group, Junshi Chen, Zhengming Chen, Robert Clarke, Rory Collins, Yu Guo, Liming Li, Jun Lv, Richard Peto, Robin Walters, Daniel Avery, Derrick Bennett, Ruth Boxall, Yumei Chang, Yiping Chen, Zhengming Chen, Robert Clarke, Huaidong Du, Simon Gilbert, Alex Hacker, Michael Holmes, Christiana Kartsonaki, Rene Kerosi, Ling Kong, Garry Lancaster, John McDonnell, Iona Millwood, Qunhua Nie, Jayakrishnan Radhakrishnan, Paul Ryder, Sam Sansome, Dan Schmidt, Rajani Sohoni, Iain Turnbull, Robin Walters, Jenny Wang, Lin Wang, Neil Wright, Ling Yang, Xiaoming Yang, Zheng Bian, Yu Guo, Xiao Han, Can Hou, Jun Lv, Pei Pei, Yunlong Tan, Canqing Yu, Zengchang Pang, Ruqin Gao, Shanpeng Li, Shaojie Wang, Yongmei Liu, Ranran Du, Yajing Zang, Liang Cheng, Xiaocao Tian, Hua Zhang, Yaoming Zhai, Feng Ning, Xiaohui Sun, Feifei Li, Silu Lv, Junzheng Wang, Wei Hou, Mingyuan Zeng, Ge Jiang, Xue Zhou, Liqiu Yang, Hui He, Bo Yu, Yanjie Li, Qinai Xu, Quan Kang, Ziyan Guo, Dan Wang, Ximin Hu, Hongmei Wang, Jinyan Chen, Yan Fu, Zhenwang Fu, Xiaohuan Wang, Min Weng, Zhendong Guo, Shukuan Wu, Yilei Li, Huimei Li, Zhifang Fu, Ming Wu, Yonglin Zhou, Jinyi Zhou, Ran Tao, Jie Yang, Jian Su, Fang Liu, Jun Zhang, Yihe Hu, Yan Lu, Liangcai Ma, Aiyu Tang, Shuo Zhang, Jianrong Jin, Jingchao Liu, Zhenzhu Tang, Naying Chen, Ying Huang, Mingqiang Li, Jinhuai Meng, Rong Pan, Qilian Jiang, Jian Lan, Yun Liu, Liuping Wei, Liyuan Zhou, Ningyu Chen, Ping Wang, Fanwen Meng, Yulu Qin, Sisi Wang, Xianping Wu, Ningmei Zhang, Xiaofang Chen, Weiwei Zhou, Guojin Luo, Jianguo Li, Xiaofang Chen, Xunfu Zhong, Jiaqiu Liu, Qiang Sun, Pengfei Ge, Xiaolan Ren, Caixia Dong, Hui Zhang, Enke Mao, Xiaoping Wang, Tao Wang, Xi Zhang, Ding Zhang, Gang Zhou, Shixian Feng, Liang Chang, Lei Fan, Yulian Gao, Tianyou He, Huarong Sun, Pan He, Chen Hu, Xukui Zhang, Huifang Wu, Pan He, Min Yu, Ruying Hu, Hao Wang, Yijian Qian, Chunmei Wang, Kaixu Xie, Lingli Chen, Yidan Zhang, Dongxia Pan, Qijun Gu, Yuelong Huang, Biyun Chen, Li Yin, Huilin Liu, Zhongxi Fu, Qiaohua Xu, Xin Xu, Hao Zhang, Huajun Long, Xianzhi Li, Libo Zhang, Zhe Qiu

**Affiliations:** Department of Epidemiology and Biostatistics, School of Public Health, Xi'an Jiaotong University Health Science Center, Xi'an, China; Medical Research Council Population Health Research Unit, Nuffield Department of Population Health, University of Oxford, Oxford, United Kingdom; Clinical Trial Service Unit and Epidemiological Studies Unit, Nuffield Department of Population Health, University of Oxford, Oxford, United Kingdom; Chinese Academy of Medical Sciences, Beijing, China; Chinese Academy of Medical Sciences, Beijing, China; Department of Epidemiology and Biostatistics, School of Public Health, Peking University Health Science Center, Beijing, China; Medical Research Council Population Health Research Unit, Nuffield Department of Population Health, University of Oxford, Oxford, United Kingdom; Clinical Trial Service Unit and Epidemiological Studies Unit, Nuffield Department of Population Health, University of Oxford, Oxford, United Kingdom; Medical Research Council Population Health Research Unit, Nuffield Department of Population Health, University of Oxford, Oxford, United Kingdom; Clinical Trial Service Unit and Epidemiological Studies Unit, Nuffield Department of Population Health, University of Oxford, Oxford, United Kingdom; Yongqinglu Community Health Service, Qingdao, Shandong Province, China; Yongqinglu Community Health Service, Qingdao, Shandong Province, China; China National Center for Food Safety Risk Assessment, Beijing, China; Department of Epidemiology and Biostatistics, School of Public Health, Peking University Health Science Center, Beijing, China; Department of Epidemiology and Biostatistics, School of Public Health, Peking University Health Science Center, Beijing, China; Medical Research Council Population Health Research Unit, Nuffield Department of Population Health, University of Oxford, Oxford, United Kingdom; Clinical Trial Service Unit and Epidemiological Studies Unit, Nuffield Department of Population Health, University of Oxford, Oxford, United Kingdom

**Keywords:** whole grains, coarse grains, cardiovascular diseases, diabetes, stroke, prospective cohort study

## Abstract

**Background:**

Lower consumption of whole grains is associated with higher risks of diabetes and coronary heart disease in Western populations, but evidence is still limited for stroke. Moreover, little is known in China, where the rates of cardiometabolic diseases are high and the grain types consumed are different from those in Western countries.

**Objectives:**

To examine the associations between coarse-grain (e.g., millet, corn, and sorghum) consumption and incident cardiometabolic diseases among Chinese adults.

**Methods:**

The prospective China Kadoorie Biobank enrolled >0.5 million adults aged 30–79 years from 10 urban and rural areas during 2004–2008. At baseline, consumption frequencies (in 5 categories from “never” to “daily”) of 12 major food groups, including coarse grains, were collected using a validated FFQ. After a median of 11 years of follow-up, 17,149 cases of diabetes, 29,876 ischemic strokes, 6097 hemorrhagic strokes, and 6704 major coronary events were recorded among 461,047 participants without a prevalence of major chronic diseases at baseline. Cox regression analyses were used to yield adjusted HRs for each disease associated with coarse-grain consumption.

**Results:**

Overall, 13.8% of participants reported regularly consuming (i.e., ≥4 days/week, regular consumers) and 29.4% reported never or rarely consuming coarse grains (i.e., nonconsumers) at baseline. Compared with nonconsumers, regular consumers had lower risks of diabetes (adjusted HR, 0.88; 95% CI, 0.78–0.98) and ischemic stroke (adjusted HR, 0.86; 95% CI, 0.81–0.93), but not hemorrhagic stroke (adjusted HR, 0.96; 95% CI, 0.76–1.20) or major coronary events (adjusted HR, 0.95; 95% CI, 0.81–1.12). For diabetes and ischemic stroke, each 100 g/day increase in the usual intake of coarse grains was associated with 14% (adjusted HR, 0.86; 95% CI, 0.76–0.97) and 13% (adjusted HR, 0.87; 95% CI, 0.81–0.94) lower risks, respectively, with similar results in various subgroups.

**Conclusions:**

In Chinese adults, higher coarse-grain consumption is associated with lower risks of diabetes and ischemic stroke, supporting the promotion of coarse-grain consumption in China.

## Introduction

Cardiometabolic diseases such as diabetes and cardiovascular disease have caused a large global health burden, accounting for >19 million annual deaths worldwide (1), including >3 million from China (2). Primary prevention of cardiometabolic diseases is therefore an important public health priority. Low consumption of whole grains has been recognized as a major modifiable risk factor for cardiometabolic diseases, responsible for an estimated 3 million deaths and 82 million disability-adjusted life-years worldwide in 2017 (3). Several large-scale meta-analyses of prospective studies have consistently reported inverse associations of whole-grain consumption with incident diabetes (4, 5) and coronary heart disease (6, 7). However, these studies were mainly conducted in Western countries, especially the United States, with limited evidence from Asian countries, including China (8, 9), where dietary habits, lifestyle factors, and disease patterns differ considerably from those in Western populations (10).

Unlike in Western countries, refined wheat and rice are major staple foods in China, followed by coarse grains, whereas whole-grain wheat and brown rice are rarely consumed (11–13). Coarse grains include grain foods (e.g., millet, corn, adlay, oats, buckwheat, and sorghum) other than wheat and rice (14) and are similar to whole grains. They are rich in dietary fiber, B vitamins, and some trace minerals, such as iron, magnesium, and zinc, when compared with refined grains (15). In addition, they contain endosperm, germ, and bran, which are classified as whole grains in Western studies and cover almost all food forms of whole grains (e.g., oats and rye) (16, 17). The Chinese Dietary Guidelines recommend that a healthy adult should consume 50–100 grams of coarse grains per day (18), but only <15% of the population reach this level at present (14). To our knowledge, evidence on the associations of coarse-grain consumption with incident cardiometabolic diseases is scare. Moreover, the associations between whole-grain consumption and stroke subtypes, especially hemorrhagic stroke, are not conclusive, underscoring the importance of conducting such studies in a population where the incidence of stroke, particularly hemorrhagic stroke, is high.

Using data from the large, nationwide China Kadoorie Biobank (CKB) prospective study, we examined the associations between coarse-grain consumption and incidences of cardiometabolic diseases, including diabetes, ischemic stroke, hemorrhagic stroke, and major coronary events, among Chinese adults. Such studies are needed for developing and refining evidence-based dietary guidelines for disease prevention in China and worldwide.

## Methods

### Study population

Details of the CKB study design, methods, and participants have been reported previously (19, 20). Briefly, the CKB is a prospective cohort study among 0.5 million adults in 10 geographical areas of China (5 urban and 5 rural). The areas were selected from China's nationally representative Disease Surveillance Points to cover diverse socioeconomic levels, disease patterns, and risk exposures. The baseline survey was conducted between June 2004 and July 2008. All permanent residents aged 35–74 years from 100–150 urban committees or rural villages in each area were invited to participate. Among them, approximately 30% responded. A total of 512,726 individuals (aged 30–79 years) were enrolled in the study, including a few slightly outside the target age range. Ethical approvals were obtained from the relevant local, national, and international ethics committees. All participants provided written informed consent prior to participation.

The present study excluded participants who had prevalent diabetes (*n* = 30,301), heart disease (*n* = 15,472), stroke (*n* = 8884), or cancer (*n* = 2578) at baseline, as well as 2 participants with missing values for BMI, leaving 461,047 participants in the final analyses.

### Data collection

Trained health workers administered laptop-based questionnaires at the local study clinics to collect information on sociodemographic factors, smoking, alcohol intake, diet, physical activity, and medical history and measured height, weight, waist circumference (WC), body fat percentage (BF%), and blood pressure following standard protocols (21, 22). BMI was calculated as weight in kilograms divided by height in meters squared. BF% was estimated by a body composition analyzer (TANITA-TBF-300 GS, Tanita). Blood pressure was measured twice using a digital sphygmomanometer (model UA-779, A&D Medical) after at least 5 minutes of rest in a seated position; the average of 2 satisfactory measurements was used. A nonfasting venous blood sample was collected for storage, and random blood glucose was measured immediately using the SureStep Plus System (Lifescan, Johnson & Johnson). Participants without a prior diabetes diagnosis but with random blood glucose levels between 7.8 and 11.1 mmol/L were invited for a fasting blood glucose test the following day. Prevalent diabetes at baseline (to be excluded from the current analysis) was defined as a measured fasting blood glucose value ≥ 7.0 mmol/L, a measured random blood glucose value ≥ 11.1 mmol/L, or a self-reported prior history of physician-diagnosed diabetes (23).

Dietary information was collected using a short, semi-quantitative FFQ, covering 12 major food groups (including white rice, wheat products, other staple foods, red meat, poultry, fish, eggs, dairy products, fresh vegetables, fresh fruit, soybean, and preserved vegetables). Participants were asked to report their habitual consumption frequencies for each food group during the previous 12 months, through choosing from the 5 frequency categories (daily, 4–6 days/week, 1–3 days/week, monthly, or never/rarely). Coarse grain was expressed as “other staple food” (i.e., a staple food other than rice and wheat products) in the FFQ, following the definition in the Chinese Resident Nutrition and Health Surveillance (14). A reproducibility and validity study of the FFQ was conducted in a subsample of 480 participants, during which 2 FFQs (median interval, 3.3 months) and twelve 24-hour dietary recalls (3 consecutive days per season) were completed by each individual. The results showed that our FFQ has satisfactory reproducibility and validity for all food groups, including coarse-grain intake, for which the weighted kappas were 0.85 for reproducibility and 0.80 for relative validity. In addition, a strong and highly significant positive association was observed between coarse-grain consumption and the blood level of acetate (SCFA, measured using NMR metabolomics) in a subsample of 4626 participants (*P* < 0.0001).

After completion of the baseline survey, about 5% participants were randomly selected for periodic resurveys; thus far, 2 resurveys had been successfully completed, in 2008 and 2013–2014. Data collection procedures in resurveys were similar to those at baseline, but included a range of new enhancements. During the second resurvey in 2013–2014, the amount of each food group consumed was recorded (in addition to the consumption frequency), which was used as a proxy to estimate the average consumption for each baseline category (10).

### Follow-up for incident cardiometabolic diseases

The vital status of participants was monitored through death registries, and checked annually against local residential and health insurance records and by active confirmation through residential administrators. In addition, information on major disease incidences and episodes of hospitalization was identified through linkage with disease registries and the national health insurance claim system. Fatal and nonfatal events were coded using the International Classification of Diseases, Tenth Edition, by trained staff blinded to the baseline information. The main outcomes in the present study were incident diabetes (E10–E14), ischemic stroke (I63), hemorrhagic stroke (I61), and major coronary events [fatal ischemic heart disease (I20–I25) plus nonfatal myocardial infarction (I21–I23)]. For the analyses of cardiovascular disease, only the first event (either stroke or major coronary events) was counted. By 1 January 2018, only 5302 (∼1%) individuals were lost to follow-up.

### Statistical analyses

Participants were categorized into 4 groups of coarse-grain consumption (≥4 days/week, 1–3 days/week, monthly, or never/rarely) to retain an adequate number of cases in each group. The prevalences and mean values of baseline characteristics were calculated in each group of coarse-grain consumption, adjusting for age, sex, and region as appropriate, using either multiple logistic regression (for binary variables) or linear regression (for continuous variables). The marginal means and 95% CIs for BMI, WC, BF%, blood glucose, and blood pressure in each group of coarse-grain consumption were estimated in men and women separately using multiple linear regression analyses with the following adjustments: age (continuous), region (10 groups), smoking (4 groups), alcohol intake (4 groups), education (4 groups), income (4 groups), total physical activity (continuous), and consumption of fresh fruit (4 groups), red meat (4 groups), and preserved vegetables (4 groups). Analyses for WC, BF%, blood glucose, and blood pressure were further adjusted for BMI (continuous).

Cox proportional hazards models were used to estimate HRs and 95% CIs for each outcome associated with coarse-grain consumption, stratified by 5-year age-at-risk, sex, and region, and adjusted for smoking, alcohol intake, education, income, BMI, total physical activity, family history of diabetes (dichotomous) or cardiovascular disease (dichotomous), and consumption of fresh fruit, red meat, and preserved vegetables. The floating absolute risk method was used to calculate the 95% CIs of each of the exposure categories, including the reference category. This method enables comparisons between any 2 exposure categories, and not only with the reference group (24). To correct for regression dilution bias, we used data from the second resurvey to estimate the mean daily usual consumption of coarse grains for each baseline category (**Supplemental Method 1; Supplemental Table 1**); in other words, the usual consumption of coarse grains was calculated by taking into account the consumption frequency at baseline and the consumption frequency and daily portion size at the second resurvey, which could be considered as the mean intake levels during the follow-up period. The HR for each 100 g/day of usual coarse-grain consumption was calculated using Cox proportional hazards models, in order to get more insights into the dose-response relationships between coarse-grain consumption and cardiometabolic disease risks. There were no missing values on variables included in the main analyses.

The proportional hazards assumption was examined by the interaction between follow-up time and coarse-grain consumption, and no violation was found. Stratified analyses were performed by baseline characteristics, and χ^2^ tests for trend and heterogeneity were performed. Sensitivity analyses were conducted by excluding the first 2 years of follow-up, excluding individuals from Henan (where 97.7% reported consuming coarse grains daily), or additional adjustments for WC, BF%, blood glucose, blood pressure, and other dietary factors. A 2-tailed *P* value < 0.05 was considered statistically significant. All analyses were conducted with SAS (version 9.3, SAS Institute). Figures were produced with R software (version 3.6.3, R Studio).

## Results

Among the 461,047 participants included in the main analysis, the mean baseline age was 51.5 years (SD, 10.5 years), 59.0% were women, and 42.3% lived in urban areas. Overall, 13.8% participants reported consumption of coarse grains regularly (i.e., ≥4 days/week; regular consumers) and 29.4% reported nonconsumption (i.e., never/rarely; nonconsumers). The average usual daily consumption of coarse grains was 31.5 g, and was higher in rural than in urban areas (40.1 g compared with 29.3 g, respectively; **Supplemental Figure 1**). But after excluding those participants from Henan, a rural area with the highest consumption of coarse grains (as over 90% of participants reported daily consumption of a traditional porridge made of corn flour), urban participants consumed coarse grains more frequently than their rural counterparts. Among the other 9 study regions (after excluding Henan), Harbin and Qingdao (2 urban areas) had the highest consumption, and Hunan and Sichuan (2 rural areas) had the lowest consumption. Participants consuming coarse grains more frequently were more likely to be women, were older, had higher education and income levels, and were less likely to be regular smokers and regular alcohol drinkers (**Table 1**). Except for red meat, consumption of other food groups was positively correlated with coarse-grain consumption.

**TABLE 1:** Baseline characteristics of the study participants according to the frequency of coarse-grain consumption among Chinese adults^1^

	Frequency of coarse-grain consumption				
Characteristics	Never/rarely	Monthly	1–3 days/week	≥4 days/week	Overall
*N*	135,449	206,794	55,407	63,397	461,047
Mean usual coarse-grain consumption,^2^ g/d	11.5	18.5	37.9	111.3	31.5
Age, y	49.8 (12.3)	50.7 (12.7)	51.8 (11.4)	55.4 (25.9)	51.5 (10.5)
Women, %	52.7	59.1	62.4	69.2	59.0
Urban residence, %	37.5	46.6	74.5	10.3	42.3
Education > 6 y, %	43.7	48.7	56.6	57.4	49.4
Household income > 20,000 yuan/y, %	36.6	42.2	49.9	50.6	42.6
Current regular smoking, %	31.8	26.9	23.6	20.0	26.8
Current regular alcohol intake, %	17.8	14.9	14.4	11.5	16.6
Family history of diabetes, %	6.1	6.3	6.6	6.7	6.3
Family history of CVD, %	19.6	19.4	21.0	21.1	19.9
Physical activity, MET-h/d	22.0 (14.3)	21.7 (14.8)	21.8 (13.2)	22.0 (20.1)	21.9 (13.9)
BMI, kg/m^2^	23.5 (3.8)	23.5 (3.9)	23.6 (3.5)	23.6 (8.0)	23.5 (3.3)
Waist circumference, cm					
Men	81.3 (11.1)	81.6 (11.4)	82.1 (9.9)	81.6 (23.9)	81.6 (9.6)
Women	78.3 (10.5)	78.5 (10.9)	78.6 (9.9)	78.6 (21.6)	78.5 (9.3)
Body fat percentage, %					
Men	21.7 (7.1)	21.9 (7.3)	22.1 (6.3)	21.7 (15.3)	21.8 (6.2)
Women	31.7 (8.1)	31.8 (8.3)	32.0 (7.6)	32.0 (16.5)	31.8 (7.0)
Random blood glucose, mmol/L	5.8 (1.3)	5.7 (1.4)	5.6 (1.2)	5.6 (2.8)	5.7 (1.1)
Systolic blood pressure, mmHg	130.3 (22.9)	130.2 (23.7)	129.8 (21.1)	128.9 (48.1)	130.0 (20.8)
Regular food consumption,^3^ %					
Fresh fruit	20.5	24.9	36.8	44.2	27.7
Red meat	47.6	48.0	49.0	40.4	47.0
Dairy	8.1	9.6	14.0	16.9	10.7
Preserved vegetables	21.6	21.4	24.6	25.8	22.5
Fresh vegetables	94.3	94.0	96.2	96.3	94.6
Eggs	19.3	22.6	28.1	33.4	23.8
Fish	7.2	8.1	10.2	14.2	8.9
Poultry	22.1	26.7	38.4	35.8	28.0
Soybean	6.7	7.7	11.6	19.5	9.5

1 Values are either the mean (SD) or percentage, and were adjusted for age, sex, and region, where appropriate. CVD, cardiovascular disease; MET, metabolic equivalent.

2 Crude mean values from 20,085 participants who attended the second resurvey and had no diabetes, cardiovascular disease, or cancer at either baseline or the second resurvey.

3 Values indicate the frequency as daily for fresh vegetable consumption, ≥1 day/week for poultry consumption, and ≥4 days/week for all other food groups.

Overall, there were no strong associations of coarse-grain consumption with the conventional cardiometabolic risk factors examined. Coarse-grain consumption was weakly and positively associated with BMI in women (but not in men) but inversely associated with BF% in men (but not in women; **Supplemental Figure 2**). After full adjustment (including the adjustment for BMI), coarse-grain consumption was weakly and inversely associated with blood pressure (in men only) and blood glucose (in both genders).

During a median follow-up of 11.2 years (∼5.0 million person-years in total), there were 17,149 incident diabetes cases, 29,876 incident ischemic strokes (including lacunar stroke), 6097 incident hemorrhagic strokes, and 6704 incident major coronary events recorded. After adjustment for all potential confounders, the risks of diabetes and ischemic stroke were inversely associated with coarse-grain consumption (*P*-trend < 0.003; **Table 2**). Compared with nonconsumers, the adjusted HRs for regular consumers were 0.88 (95% CI, 0.78–0.98) for diabetes and 0.86 (95% CI, 0.81–0.93) for ischemic stroke. After correcting for regression dilution bias, each 100 g/day increase in usual coarse-grain consumption was associated with a 14% lower risk of diabetes (adjusted HR, 0.86; 95% CI, 0.76–0.97) and a 13% lower risk of ischemic stroke (adjusted HR, 0.87; 95% CI, 0.81–0.94). However, there were no clear associations of coarse-grain consumption with hemorrhagic stroke and major coronary events (Table 2). Adjusted HRs and 95% CIs without using the floating absolute risk methods are presented in the **Supplemental Table 2**.

**TABLE 2:** Risks of incident cardiometabolic diseases associated with coarse-grain consumption among Chinese adults^1^

		HR (95% CI)		
Coarse-grain consumption	Events, *n*	Model 1	Model 2	Model 3
Diabetes				
Never/rarely	6340	1.00 (0.97–1.03)	1.00 (0.97–1.03)	1.00 (0.97–1.03)
Monthly	7807	0.96 (0.94–0.98)	0.96 (0.94–0.97)	0.95 (0.93–0.97)
1–3 d/week	1839	0.95 (0.91–0.99)	0.93 (0.88–0.98)	0.93 (0.88–0.98)
≥4 d/week	1163	0.89 (0.79–0.99)	0.87 (0.77–0.98)	0.88 (0.78–0.98)
Likelihood ratio chi-square^2^	—	20.71	11.62	10.79
*P-*trend	—	0.0001	0.0005	0.0007
Per 100 g/d at baseline^3^	17149	0.91 (0.83–1.00)	0.87 (0.79–0.96)	0.88 (0.80–0.97)
Per 100 g/d usual consumption^4^	17149	0.89 (0.79–1.00)	0.85 (0.75–0.96)	0.86 (0.76–0.97)
Ischemic stroke				
Never/rarely	6740	1.00 (0.97–1.03)	1.00 (0.97–1.03)	1.00 (0.97–1.03)
Monthly	11644	0.98 (0.96–0.99)	0.98 (0.96–1.00)	0.98 (0.96–1.00)
1–3 d/week	4865	0.95 (0.92–0.98)	0.96 (0.93–0.99)	0.97 (0.94–1.00)
≥4 d/week	6627	0.83 (0.78–0.89)	0.85 (0.79–0.91)	0.86 (0.81–0.93)
Likelihood ratio chi-square^2^	—	24.90	20.52	14.77
*P-*trend	—	<0.0001	<0.0001	0.002
Per 100 g/d at baseline^3^	29876	0.87 (0.82–0.92)	0.88 (0.83–0.93)	0.90 (0.85–0.95)
Per 100 g/d usual consumption^4^	29876	0.84 (0.78–0.90)	0.85 (0.79–0.91)	0.87 (0.81–0.94)
Hemorrhagic stroke				
Never/rarely	1712	1.00 (0.95–1.05)	1.00 (0.95–1.05)	1.00 (0.95–1.06)
Monthly	2836	0.92 (0.89–0.96)	0.94 (0.91–0.98)	0.94 (0.91–0.98)
1–3 d/week	623	0.94 (0.86–1.02)	0.98 (0.90–1.06)	0.99 (0.91–1.08)
≥4 d/week	926	0.89 (0.71–1.12)	0.94 (0.75–1.18)	0.96 (0.76–1.20)
Likelihood ratio chi-square^2^	—	6.62	3.61	3.58
*P-*trend	—	0.05	0.27	0.47
Per 100 g/d at baseline^3^	6097	0.91 (0.77–1.09)	0.96 (0.81–1.15)	0.99 (0.84–1.18)
Per 100 g/d usual consumption^4^	6097	0.89 (0.72–1.11)	0.95 (0.77–1.18)	0.98 (0.79–1.22)
Major coronary events				
Never/rarely	1539	1.00 (0.95–1.05)	1.00 (0.95–1.05)	1.00 (0.95–1.06)
Monthly	3037	1.03 (0.99–1.07)	1.05 (0.98–1.13)	1.07 (0.98–1.17)
1–3 d/week	1035	1.03 (0.96–1.10)	1.07 (0.97–1.18)	1.09 (0.97–1.23)
≥4 d/week	1093	0.89 (0.76–1.05)	0.94 (0.80–1.10)	0.95 (0.81–1.12)
Likelihood ratio chi-square^2^	—	3.61	4.95	7.06
*P-*trend	—	0.90	0.62	0.53
Per 100 g/d at baseline^3^	6704	0.93 (0.82–1.06)	0.98 (0.86–1.12)	0.99 (0.87–1.13)
Per 100 g/d usual consumption^4^	6704	0.91 (0.77–1.07)	0.96 (0.82–1.13)	0.98 (0.83–1.15)

1 Model 1 was stratified by age-at-risk, sex, and region. Model 2 was adjusted for the same variables as in Model 1, and additionally adjusted for education, income, smoking, alcohol intake, BMI, total physical activity, and family history of diabetes or cardiovascular disease. Model 3 was adjusted for the same variables as in Model 2, and additionally adjusted for the consumption of fresh fruit, meat, and preserved vegetables.

2 The likelihood ratio chi-square (χ^2^) values indicate the strength of the association of coarse-grain consumption with cardiometabolic diseases. A larger χ^2^ value indicates a stronger association, and a decrease in the χ^2^ value indicates that the association is attenuated after an additional adjustment for newly added variables.

3 Baseline consumption was estimated using daily consumption in the second resurvey multiplied by the consumption frequency at baseline.

4 The mean amount consumed at the second resurvey was used to estimate the usual consumption level for each group.

**FIGURE 1:**
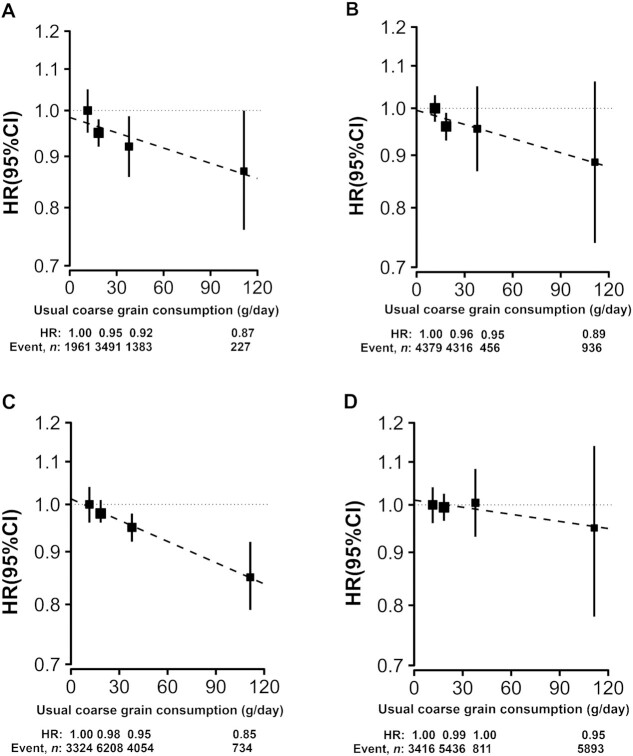
Adjusted HRs (95% CIs) for (A) incident diabetes in urban areas, (B) incident diabetes in rural areas, (C) incident ischemic stroke in urban areas, and (D) incident ischemic stroke in rural areas associated with coarse-grain consumption among Chinese adults. Analyses were stratified by age-at-risk, region, and sex, and were adjusted for education, income, smoking, alcohol intake, BMI, total physical activity, family history of diabetes or cardiovascular disease, and consumption of fresh fruit, red meat, and preserved vegetables. The black boxes indicate HRs, with the sizes inversely proportional to the variance of the logarithm of the HR, and the corresponding mean usual coarse-grain consumptions for each category were 12, 19, 38, and 113 g/day (for panels A–D, respectively). The vertical lines indicate 95% CIs.

**FIGURE 2:**
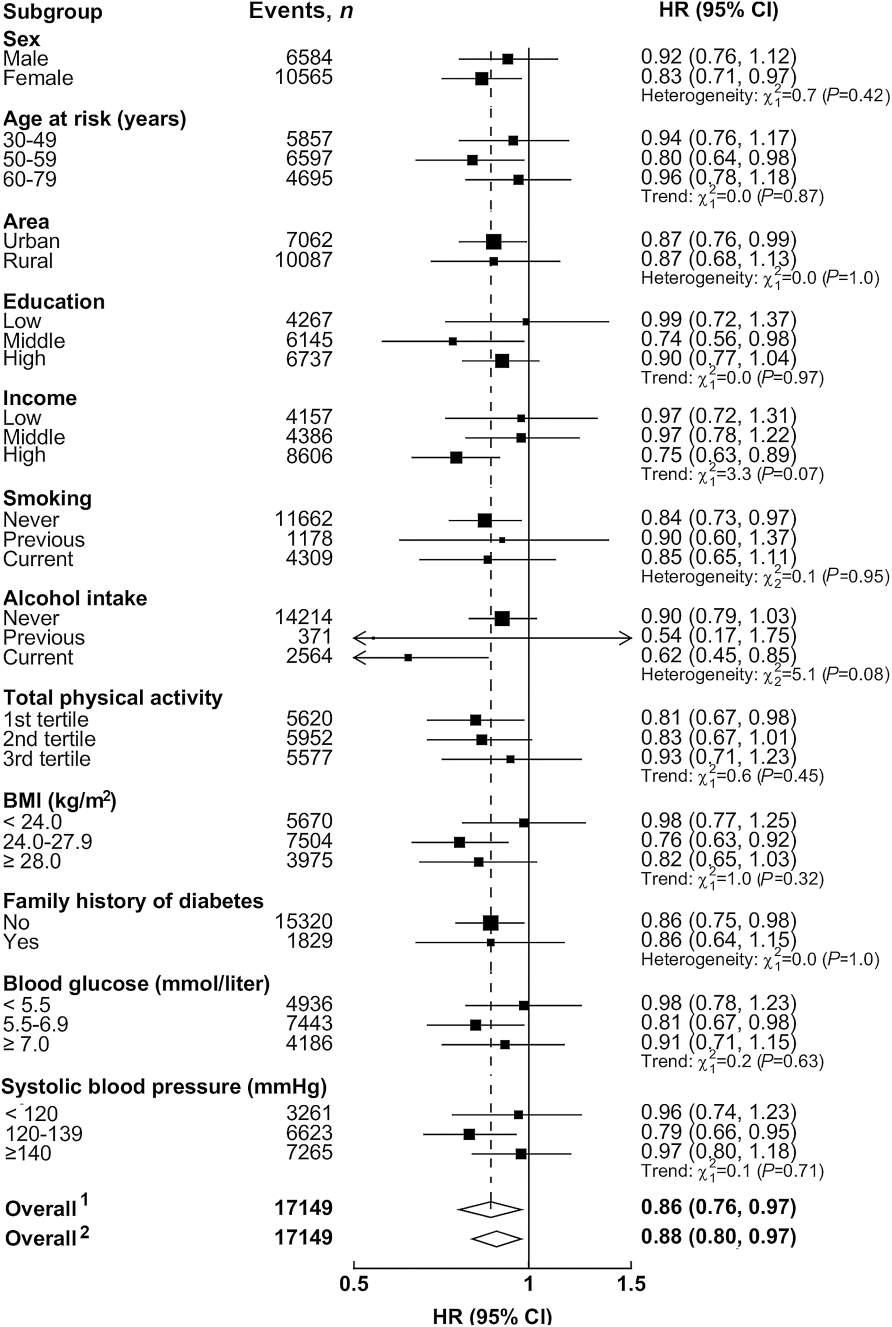
Adjusted HRs (95% CIs) for incident diabetes per 100 g/day of coarse-grain consumption by subgroups among Chinese adults. Analyses were stratified by age-at-risk, region, and sex, and adjusted for education, income, smoking, alcohol intake, BMI, total physical activity, family history of diabetes, and consumption of fresh fruit, red meat, and preserved vegetables. The black boxes represent HRs, with the size inversely proportional to the variance of the logarithm of the HR, and the horizontal lines represent 95% CIs. ^1^Overall HR per 100 g/day usual coarse grain consumption after correction for regression dilution bias. ^2^Overall HR per 100 g/day usual coarse grain consumption before correction for regression dilution bias.

**FIGURE 3:**
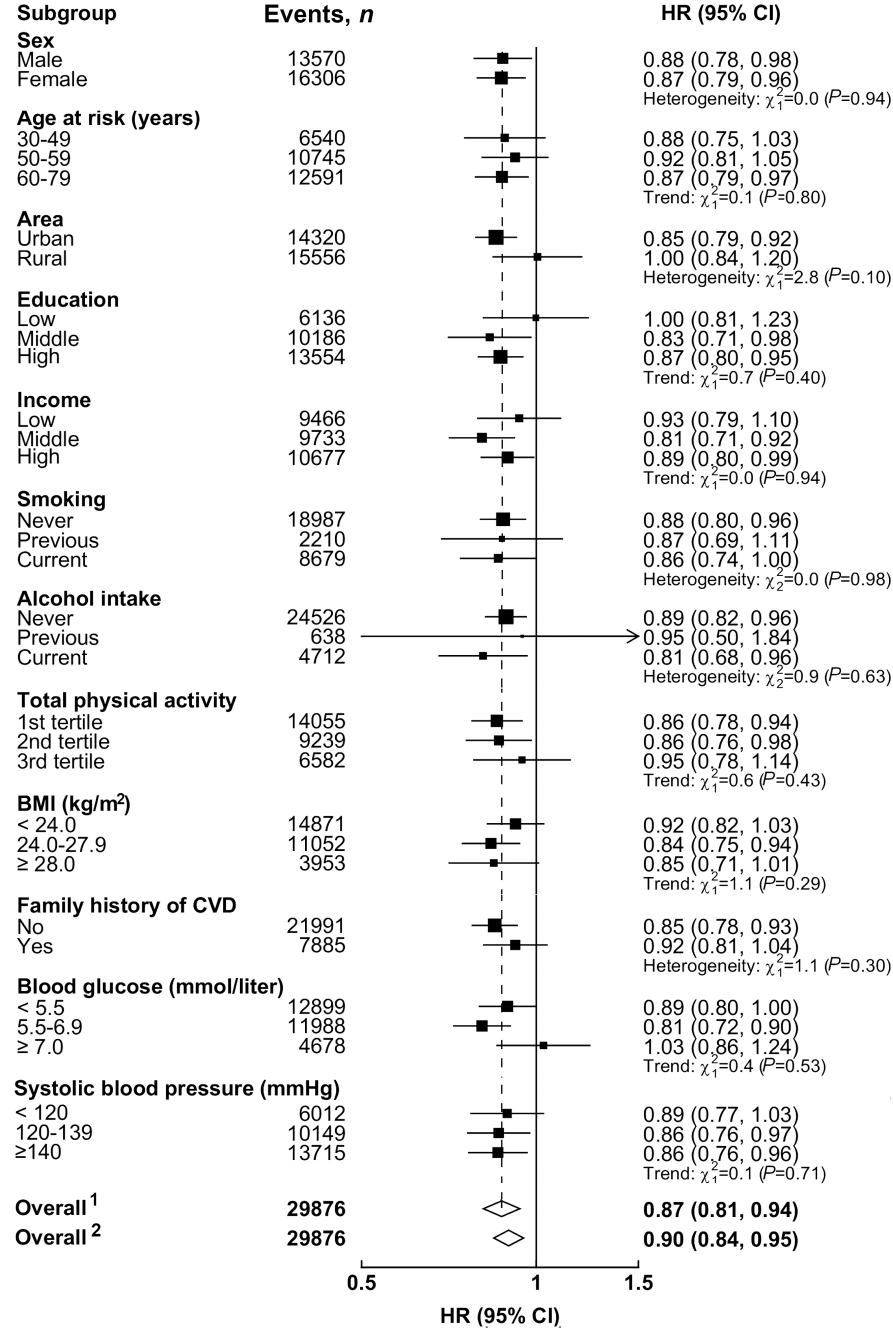
Adjusted HRs (95% CIs) for incident ischemic stroke per 100 g/day of coarse-grain consumption by subgroups among Chinese adults. Analyses were stratified by age-at-risk, region, and sex, and adjusted for education, income, smoking, alcohol intake, BMI, total physical activity, family history of cardiovascular disease, and consumption of fresh fruit, red meat, and preserved vegetables. The black boxes represent HRs, with the size inversely proportional to the variance of the logarithm of the HR, and the horizontal lines represent 95% CIs. CVD, cardiovascular disease. ^1^Overall HR per 100 g/day usual coarse grain consumption after correction for regression dilution bias. ^2^Overall HR per 100 g/day usual coarse grain consumption before correction for regression dilution bias.

The association of coarse-grain consumption with diabetes was rather similar in urban and rural areas, with adjusted HRs of 0.87 (95% CI, 0.76–1.00) and 0.89 (95% CI, 0.74–1.06), respectively, when comparing regular and no consumption (**Figure 1**A and B). However, the association with ischemic stroke appeared to be somewhat stronger in urban than in rural area [0.85 (95% CI, 0.79–0.92) compared with 0.95 (95% CI, 0.78–1.14), Figure 1C and D]. These associations were not significantly modified by baseline characteristics, including sex, age, education and income levels, smoking and alcohol status, total physical activity, and BMI (**Figures 2** and **3**). There was no substantial heterogeneity for the associations in 5 urban and 5 rural areas either (**Supplemental Figure 3**). In sensitivity analyses excluding the first 2 years of follow-up, excluding individuals from Henan, or additional adjustments for WC, BF%, blood glucose, blood pressure, and other dietary factors, the associations were not materially altered (**Supplemental Table 3**).

## Discussion

This large, prospective study of Chinese adults showed that higher coarse-grain consumption was associated with lower incidences of diabetes and ischemic stroke, with each 100 g/day increase in the usual intake associated with 14% and 13% lower risks, respectively. These associations were largely similar in various subgroups of baseline characteristics and across the 10 regions. However, we did not observe any significant association of coarse-grain consumption with hemorrhagic stroke and major coronary events.

A number of previous studies in Western populations have reported an inverse association of whole-grain consumption with the risk of diabetes. In a recent meta-analysis of 13 prospective studies involving 29,633 diabetes cases, each additional 30 g of daily whole-grain consumption was associated with a 13% (7%–18%) lower risk of diabetes (5), similar to the risk estimate for each 100 g/day of coarse-grain consumption observed in the present study. In that meta-analysis, however, there was great heterogeneity in the risk estimates across different geographic locations and by methods of dietary assessment (5). In different populations, whole grains may be consumed or defined differently (i.e., including different types of grain foods and calculated at the grain, ingredient, and food levels), making direct quantitative comparisons of study findings difficult. The coarse grains in the present study are grains other than wheat products and rice (which are usually refined), with millet and corn being the most frequently consumed types (14). In contrast, the whole grains typically consumed in Western populations mainly include whole-grain wheat, oats, rye, barley, and brown rice (16, 17), which are rarely consumed in China (11–13). The coarse grains consumed in China may have different potential impacts on health compared with the whole grains consumed by Western populations, due to the variations in their nutritional profiles and glycemic properties (25). For example, millet and corn, the most common types of coarse grains in China, have lower levels of dietary fiber and bioactive compounds and higher glycemic index values compared with whole-grain wheat, oats, and rye (25).

Prospective studies on whole-grain consumption and incident stroke are limited, especially for hemorrhagic stroke, which is relatively uncommon in Western populations. A meta-analysis with 3 prospective studies, involving 1191 stroke cases in Western populations (7), and the Prospective Urban and Rural Epidemiology (PURE) study, involving 3227 stroke cases across 21 countries (26), both reported a null association between whole-grain consumption and total stroke, but no data were presented separately for stroke subtypes and no separate analysis among participants from Asian countries, including China, was conducted, possibly because of the limited numbers of cases (26). However, some studies have reported similar findings as ours. For example, a more recent combined analysis of data from 2 US cohort studies involving 2458 ischemic stroke cases showed that consumption of whole-grain, cold breakfast cereal and bran was significantly and inversely associated with ischemic stroke, with HRs similar to those observed in the present study (17). In China, a recent case-control study of 990 ischemic stroke cases and 990 controls reported a significantly inverse association between the plasma biomarker of whole-grain intake—alkylresorcinol metabolite 3-(3,5-dihydroxyphenyl)-1-propanoic acid—and the ischemic stroke risk (9).

In terms of hemorrhagic stroke, previously only 1 small, prospective study in Finland, involving just 58 cases, has been conducted, reporting no clear association between whole-grain consumption and the risk of hemorrhagic stroke (27). The present study included a much large number of well-characterized hemorrhagic stroke cases, but did not find any significant association for coarse grains either.

The null association between coarse-grain consumption and major coronary events in the present study was consistent with the latest evidence from the PURE study, which involved 137,130 eligible participants in low-, medium-, and high-income countries (including 41,596 participants from China) (26). However, a previous meta-analysis of 5 prospective studies involving 6557 coronary heart disease cases (mainly in the United States) reported a strong, inverse association between whole-grain consumption and incident coronary heart disease (HR, 0.84; 95%CI, 0.77-0.92, per 90 g/day) (7). The reasons underlying the different results are not clear to us, but this emphasizes the necessity of conducting further studies to confirm or refute the present study findings.

Although at present the CKB data do not allow us to further examine the biological mechanisms underlying those observed associations, based on the literature, some nutrients rich in coarse grains may be involved. For example, coarse grains are rich sources of fermentable carbohydrates, including dietary fiber, resistant starch, and oligosaccharides (15), which can be fermented to SCFAs, such as acetate, butyrate, and propionate, in the colon (28). As in our study, coarse-grain consumption was positively associated with the blood level of acetate, which was the only SCFA measured in the CKB thus far. The increased production of an SCFA can help lower serum cholesterol and improve glucose homeostasis (29). The relatively rich dietary fiber in coarse grains may also slow digestion and absorption and improve postprandial glucose and insulin responses, thus leading to better glycemic control (30). Other bioactive compounds, including phytic acid, carotenoids, and phenolic compounds, that are present in coarse grains may also contribute to cardiometabolic health by acting on the oxidative stress and systemic inflammation (31–33).

The present study is the first and largest prospective investigation of the associations of coarse-grain consumption with incidences of diabetes and stroke in Asian countries. Apart from the large sample size and prospective cohort design, the chief strengths of the present study include the well-characterized disease event cases (e.g., >90% imaging-confirmed stroke cases), careful adjustment for potential factors and regression dilution bias, and good reproducibility and validity of the FFQ. However, this study also has some limitations. First, we did not collect information on the consumption types of coarse grains. Individual whole-grain foods may have heterogeneous effects on health outcomes because of the variations in nutritional composition, although most previous studies have reported largely consistent results about the effects of whole-grain subtypes on diabetes and cardiovascular disease (4, 7). According to the Chinese Residents Nutrition and Health Surveillance, the most frequently consumed coarse grains in China are millet and corn (14). Second, the FFQ at baseline did not include the consumption amounts of food groups, including coarse grains (i.e., only the consumption frequency was collected). Therefore, the usual amounts of coarse-grain consumption during follow-up were estimated indirectly from the second resurvey, assuming the daily portion sizes on the days when participants consumed coarse grains did not change from baseline to the second resurvey. We used these group mean levels of usual consumption amounts (i.e., a same value was assigned to every individual participant in the same baseline consumption frequency group) to estimate the linear associations between coarse-grain intake and risks of cardiometabolic diseases to facilitate the comparison with results from other studies. Although the same estimation method has been applied in other large, prospective cohort studies, such as the Million Women Study (34) and the UK Biobank (35), the actual CIs of the linear associations could be slightly wider. Thirdly, intakes of total energy and some specific nutrients (e.g., dietary fiber) could not be taken into account in the analyses due to the lack of data. However, the total energy intake should not have a major confounding role in our findings, because we adjusted our analyses for both BMI and total physical activity, which together could be a good proxy for total energy intake (36). Fourthly, most of the incident diabetes cases recorded in our study were of an unspecified type. Previously, it has been reported that the incidence rate of type 1 diabetes was about 0.51 per 100,000 person years among Chinese adults over 30 years old (37). Therefore, it is reasonable to assume that only a small number of incident diabetes cases were of type 1 diabetes, because of the ages of our study population (i.e., >30 years at baseline). Finally, due to the observational nature of the study, we cannot exclude the possibility of residual confounding or confirm the causality of the observed associations.

In summary, in this large sample of Chinese adults, higher consumption of coarse grains was associated with lower risks of diabetes and ischemic stroke. Although further studies are warranted to confirm (or refute) the associations and to elucidate the potential underlying mechanisms, our findings do suggest that promoting increased consumption of coarse grains (i.e., to replace refined grains) in our population might be an effective public health strategy for the prevention of cardiometabolic diseases in China.

The China Kadoorie Biobank Collaborative Group includes members of the:

- International Steering Committee: Junshi Chen, MD, Zhengming Chen, DPhil (principal investigator), Robert Clarke, FRCP, Rory Collins, FRS, Yu Guo, MSc, Liming Li, PhD (principal investigator), Jun Lv, PhD, Richard Peto, FRS, and Robin Walters, PhD;
- International Coordinating Centre, Oxford, UK: Daniel Avery, MSc, Derrick Bennett, PhD, Ruth Boxall, MD, Yumei Chang, PhD, Yiping Chen, DPhil, Zhengming Chen, DPhil, Robert Clarke, FRCP, Huaidong Du, PhD, Simon Gilbert, MSc, Alex Hacker, BA, Michael Holmes, PhD, Christiana Kartsonaki, DPhil, Rene Kerosi, MSc, Ling Kong, BSc, Garry Lancaster, BA, John McDonnell, GD (graduation diploma) (Comp SC: Computer Science), Iona Millwood, DPhil, Qunhua Nie, BA, Jayakrishnan Radhakrishnan, MSc, Paul Ryder, BA, Sam Sansome, BSc, Dan Schmidt, MSc, Rajani Sohoni, BSc, Iain Turnbull, MBBS, Robin Walters, PhD, Jenny Wang, MSc, Lin Wang, MSc, Neil Wright, MSc, Ling Yang, PhD, and Xiaoming Yang, PhD; and
- National Coordinating Centre, Beijing, China: Zheng Bian, MD, Yu Guo, MSc, Xiao Han, BA, Can Hou, BSc, Jun Lv, PhD, Pei Pei, BSc, Yunlong Tan, MD, and Canqing Yu, PhD.

The Group had contributors from 10 Regional Coordinating Centers:

- Qingdao:
  - Qingdao CDC: Zengchang Pang, Ruqin Gao, Shanpeng Li, Shaojie Wang, Yongmei Liu, Ranran Du, Yajing Zang, Liang Cheng, Xiaocao Tian, Hua Zhang, Yaoming Zhai, Feng Ning, Xiaohui Sun, and Feifei Li.
  - Licang CDC: Silu Lv, Junzheng Wang, and Wei Hou.
- Harbin:
  - Heilongjiang Provincial CDC: Mingyuan Zeng, Ge Jiang, and Xue Zhou.
  - Nangang CDC: Liqiu Yang, Hui He, Bo Yu, Yanjie Li, Qinai Xu, Quan Kang, and Ziyan Guo.
- Haikou:
  - Hainan Provincial CDC: Dan Wang, Ximin Hu, Hongmei Wang, Jinyan Chen, Yan Fu, Zhenwang Fu, and Xiaohuan Wang.
  - Meilan CDC: Min Weng, Zhendong Guo, Shukuan Wu, Yilei Li, Huimei Li, and Zhifang Fu.
- Suzhou:
  - Jiangsu Provincial CDC: Ming Wu, Yonglin Zhou, Jinyi Zhou, Ran Tao, Jie Yang, and Jian Su.
  - Suzhou CDC: Fang Liu, Jun Zhang, Yihe Hu, Yan Lu, Liangcai Ma, Aiyu Tang, Shuo Zhang, Jianrong Jin, and Jingchao Liu.
- Liuzhou:
  - Guangxi Provincial CDC: Zhenzhu Tang, Naying Chen, and Ying Huang.
  - Liuzhou CDC: Mingqiang Li, Jinhuai Meng, Rong Pan, Qilian Jiang, Jian Lan, Yun Liu, Liuping Wei, Liyuan Zhou, Ningyu Chen, Ping Wang, Fanwen Meng, Yulu Qin, and Sisi Wang.
- Sichuan:
  - Sichuan Provincial CDC: Xianping Wu, Ningmei Zhang, Xiaofang Chen, and Weiwei Zhou.
  - Pengzhou CDC: Guojin Luo, Jianguo Li, Xiaofang Chen, Xunfu Zhong, Jiaqiu Liu, and Qiang Sun.
- Gansu:
  - Gansu Provincial CDC: Pengfei Ge, Xiaolan Ren, and Caixia Dong.
  - Maiji CDC: Hui Zhang, Enke Mao, Xiaoping Wang, Tao Wang, and Xi Zhang.
- Henan:
  - Henan Provincial CDC: Ding Zhang, Gang Zhou, Shixian Feng, Liang Chang, and Lei Fan.
  - Huixian CDC: Yulian Gao, Tianyou He, Huarong Sun, Pan He, Chen Hu, Xukui Zhang, Huifang Wu, and Pan He.
- Zhejiang:
  - Zhejiang Provincial CDC: Min Yu, Ruying Hu, and Hao Wang.
  - Tongxiang CDC: Yijian Qian, Chunmei Wang, Kaixu Xie, Lingli Chen, Yidan Zhang, Dongxia Pan, and Qijun Gu.
- Hunan:
  - Hunan Provincial CDC: Yuelong Huang, Biyun Chen, Li Yin, Huilin Liu, Zhongxi Fu, and Qiaohua Xu.
  - Liuyang CDC: Xin Xu, Hao Zhang, Huajun Long, Xianzhi Li, Libo Zhang, and Zhe Qiu.

## Data Availability

Data described in the manuscript, code book, and analytic code will be made available upon request pending application and approval.

## Supplementary Material

nxac041_Supplemental_File

## Notes

The China Kadoorie Biobank study is jointly coordinated by the University of Oxford and the Chinese Academy of Medical Sciences. Funding resources include grants 2016YFC0900500, 2016YFC0900501, 2016YFC0900504, and 2016YFC1303904 from the National Key Research and Development Program of China; grants 202922/Z/16/Z, 104085/Z/14/Z, and 088158/Z/09/Z from the UK Wellcome Trust; grants 81390540, 81390541, and 81390544 from the Chinese National Natural Science Foundation; and core funding provided by the British Heart Foundation, UK Medical Research Council, and Cancer Research United Kingdom to the Clinical Trial Service Unit and Epidemiological Studies Unit, University of Oxford. The baseline survey of the China Kadoorie Biobank is funded by the Kadoorie Charitable Foundation, Hong Kong, China. Jiaomei Yang acknowledges support from the China Scholarship Council (201906285048).

Author disclosures: The authors report no conflicts of interest.

The supporting sources had no involvement in study design; collection, analysis, and interpretation of data; writing of the report; or any restrictions regarding the submission of the report for publication.

Supplemental Method 1, Supplemental Tables 1–3, and Supplemental Figures 1–3 are available from the “Supplementary data” link in the online posting of the article and from the same link in the online table of contents at https://academic.oup.com/jn/.

Abbreviations used: BF%, body fat percentage; CKB, China Kadoorie Biobank; PURE, Prospective Urban and Rural Epidemiology; WC, waist circumference.
