# Supplementary material for: Coarse Grain Consumption and Risk of Cardiometabolic Diseases: A Prospective Cohort Study of Chinese Adults
Source: J Nutr. 2022 Mar 2;152(6):1476–86. doi: 10.1093/jn/nxac041 (PMC9178969; doi:10.1093/jn/nxac041)
Supplement: nxac041_Supplemental_File [file nxac041_supplemental_file.docx]

**Supplementary materials** (1 Methods, 3 Tables, and 3 Figures)

**Supplemental Method 1 Calculation of the usual amount of coarse grain consumption.**

**Supplemental Table 1. Baseline and usual consumption per day for each baseline category of coarse grain consumption based on 20,085 participants attending the second resurvey in 2013-2014 among Chinese adults.**

**Supplemental Table 2. Adjusted hazard risks for incident cardiometabolic diseases according to coarse grain consumption without using floating absolute risk method among Chinese adults.**

**Supplemental Table 3. Adjusted HRs for incident cardiometabolic diseases per 100 g/day of coarse grain consumption with different exclusions and adjustments among Chinese adults.**

**Supplemental Figure 1.** **Distribution and mean value of coarse grain consumption by region at resurvey in 2013-2014 among Chinese adults.** The bar graph indicates the distribution of coarse grain consumption, with y axis on the left. The point graph indicates the mean consumption of coarse grains, with y axis on the right.

**Supplemental Figure 2.** **Adjusted mean values for A) body mass index (BMI), B) body fat percentage, C) random blood glucose, and D) systolic blood pressure by the frequency of coarse grain consumption among Chinese adults.** Mean values for BMI were adjusted for age, region, education, income, smoking, alcohol intake, total physical activity, and consumption of fresh fruit, red meat and preserved vegetables. Mean values for body fat percentage, random blood glucose and systolic blood pressure were additionally adjusted for BMI. **P* for trend <0.005.

**Supplemental Figure 3. Adjusted HRs (95%CIs) for incident diabetes and ischemic stroke per 100 g/day of coarse grain consumption by region among Chinese adults.** Analyses were stratified by age-at-risk, region and sex, and adjusted for education, income, smoking, alcohol intake, BMI, total physical activity, family history of diabetes or cardiovascular disease, and consumption of fresh fruit, red meat and preserved vegetables. The black boxes represent hazard ratios, with the size inversely proportional to the variance of the logarithm of the hazard ratio, and the horizontal lines represent 95%CI.

**Supplemental Method 1. Calculation of the usual amount of coarse grain consumption**

The group mean level of usual amount of coarse grain consumption (in grams per day) was calculated for each baseline consumption group, using the following steps:

1. The mean daily portions on the days when participants consumed coarse grains for each of the four coarse grain consumption groups namely “never/rarely”, “1-3 days/month”, “1-3 days/week”, or “≥4 days/week” were computed using data collected during the second resurvey. These were found to be 0 for the “never/rarely” group, 134.46 g for the “1-3 days/month” group, 135.58 g for the “1-3 days/week” group, and 149.75 g for the “≥4 days/week” group.

2. Assuming this daily portion size was the same at the baseline (i.e. people tend to change their frequency of food consumption but the daily portion size remains roughly constant).

3. The average amounts of daily coarse grain consumption at baseline were derived using the mean daily portions multiplied by a constant number assigned to each group, which was 0 for the “never/rarely” group, 2/30 days for the “monthly” group, 2/7 days for the “1-3 days/week” group, and 5.5/7 days for the “≥4 days/week” group. The derived baseline average amounts of daily coarse grain consumption was 0, 8.98 g, 38.74 g, and 117.66 g, respectively, for “never/rarely”, “monthly”, “1-3 days/week”, and “≥4 days/week” coarse grain consumption groups.

4. Taking into account the consumption frequency variation from the baseline to the second resurvey, which was conducted at roughly the mid-point of the follow-up period, the mean “**usual amount of coarse grain consumption**” for each baseline group was calculated by summing up the products of multiplying the “**baseline amount of coarse grain consumption**” for each group (calculated in the Step 3 above) by the percentage of participants in each of the consumption frequency group at the second resurvey. For example, for the baseline “never/rarely” group, at the second resurvey 56.80% remained at the “never/rarely” group, 26.93% switched to the “monthly” group, 12.81% switched to the “1-3 days/week” group, and 3.46% switched to the “≥4 days/week” group. The usual amount of coarse grain consumption for the baseline “never/rarely” group was therefore equal to 56.80%*0 + 26.93%*8.98 + 12.81%*38.74 + 3.46%*117.66 = 11.45 grams per day (see Supplemental Table 1 on next page).

5. The usual amounts of coarse grain consumption for the other baseline categories of coarse grain consumption were calculated using the same approach, as 18.45, 37.86, and 111.34 grams per day respectively. This group mean usual amount of coarse grain consumption was assigned to each individual participant in the corresponding baseline group in order to estimate the linear association of per 100 g/day of coarse grain consumption with study outcomes.

**Supplemental Table 1. Baseline and usual consumption per day for each baseline category of coarse grain consumption based on 20,085 participants attending the second resurvey in 2013-2014 among Chinese adults.**

|  | Baseline  ***n*** | No. of participants | 2^nd^ resurvey (mean 8.0 years later)  **F** | | | | Consumption days^1^ | Mean daily  portion (g)^2^ | Baseline consumption (g/d)  **B** | Usual consumption (g/d)^3^  **U** |
| --- | --- | --- | --- | --- | --- | --- | --- | --- | --- | --- |
|  |  |  | Never/rarely  1 | Monthly  2 | 1-3 days/week  3 | ≥ 4 days/week  4 |  |  |  |  |
| 1 | Never/rarely | 8084 | 56.80% | 26.93% | 12.81% | 3.46% | 0 | 0 | B_1_=0 | U_1_=11.45 |
| 2 | Monthly | 4582 | 43.85% | 26.93% | 23.25% | 5.97% | 0.07 | 134.46 | B_2_=8.98  (134.46×0.07) | U_2_=18.45 |
| 3 | 1-3 days/week | 3800 | 23.86% | 20.50% | 37.31% | 18.33% | 0.29 | 135.58 | B_3_=38.74  (135.58×0.29) | U_3_=37.86 |
| 4 | ≥4 days/week | 3619 | 1.74% | 0.98% | 4.08% | 93.21% | 0.79 | 149.75 | B_4_=117.66  (149.75×0.79) | U_4_=111.34 |

^1^ The number of consumption days for each baseline category were 0, 2/30 (0.07), 2/7 (0.29), and 5.5/7 (0.79), respectively.

^2^ The mean daily portion was the average portion size on the days when participants consumed coarse grains from the second survey, used as a proxy of baseline mean daily portion.

^3^ Usual intake amount for each group was estimated by taking into account changes in consumption frequency between baseline and the second resurvey using this formula U_n_ = $\sum_{i=1}^{4} (F\mathrm{ni} \times Bi)$; F is the percentage in each cell, B is the baseline consumption per day for each baseline category, U is the usual consumption per day for each baseline category.

**Supplemental Table 2. Adjusted hazard ratios for incident cardiometabolic diseases according to coarse grain consumption without using floating absolute risk method among Chinese adults.**

| **Coarse grain consumption** | **Events, *n*** | **HR (95%CI)** | | |
| --- | --- | --- | --- | --- |
|  |  | **Model 1^1^** | **Model 2^2^** | **Model 3^3^** |
| **Diabetes** |  |  |  |  |
| Never/rarely | 6340 | 1.00 | 1.00 | 1.00 |
| Monthly | 7807 | 0.96 (0.93-0.99) | 0.96 (0.92-0.99) | 0.95 (0.92-0.98) |
| 1-3 days/week | 1839 | 0.95 (0.91-1.01) | 0.93 (0.88-0.98) | 0.93 (0.88-0.98) |
| ≥4 days/week | 1163 | 0.89 (0.79-1.02) | 0.87 (0.76-0.99) | 0.88 (0.77-0.99) |
| **Ischemic stroke** |  |  |  |  |
| Never/rarely | 6740 | 1.00 | 1.00 | 1.00 |
| Monthly | 11644 | 0.98 (0.95-1.01) | 0.98 (0.95-1.01) | 0.98 (0.95-1.01) |
| 1-3 days/week | 4865 | 0.95 (0.91-0.99) | 0.96 (0.92-0.99) | 0.97 (0.93-1.01) |
| ≥4 days/week | 6627 | 0.83 (0.77-0.90) | 0.85 (0.79-0.91) | 0.86 (0.80-0.93) |
| **Hemorrhagic stroke** |  |  |  |  |
| Never/rarely | 1712 | 1.00 | 1.00 | 1.00 |
| Monthly | 2836 | 0.92 (0.87-0.98) | 0.94 (0.88-1.00) | 0.94 (0.89-1.00) |
| 1-3 days/week | 623 | 0.94 (0.85-1.04) | 0.98 (0.88-1.08) | 0.99 (0.90-1.10) |
| ≥4 days/week | 926 | 0.89 (0.71-1.13) | 0.94 (0.74-1.19) | 0.96 (0.76-1.21) |
| **Major coronary events** |  |  |  |  |
| Never/rarely | 1539 | 1.00 | 1.00 | 1.00 |
| Monthly | 3037 | 1.03 (0.97-1.09) | 1.05 (0.99-1.12) | 1.07 (0.99-1.15) |
| 1-3 days/week | 1035 | 1.03 (0.94-1.12) | 1.07 (0.98-1.17) | 1.09 (0.99-1.20) |
| ≥4 days/week | 1093 | 0.89 (0.75-1.06) | 0.94 (0.79-1.12) | 0.95 (0.80-1.14) |

^1^ Model 1: stratified by age-at-risk, sex and region.

^2^ Model 2: as for model 1, additionally adjusted for education, income, smoking, alcohol intake, BMI, total physical activity, family history of diabetes or cardiovascular disease.

^3^ Model 3: as for model 2, additionally adjusted for the consumption of fresh fruit, meat, and preserved vegetables.

**Supplemental Table 3. Adjusted HRs for incident cardiometabolic diseases per 100 g/day of coarse grain consumption with different exclusions and adjustments among Chinese adults^1^.**

|  | **Diabetes** | **Ischemic stroke** | **Hemorrhagic stroke** | **Major coronary events** |
| --- | --- | --- | --- | --- |
| **Excluding the first two years follow-up** | 0.86 (0.76-0.97) | 0.87 (0.81-0.94) | 0.98 (0.79-1.22) | 0.98 (0.83-1.15) |
| **Excluding participants from Henan** | 0.86 (0.76-0.97) | 0.87 (0.81-0.94) | 0.98 (0.79-1.22) | 0.98 (0.83-1.15) |
| **Additional adjustment** |  |  |  |  |
| **+ waist circumference** | 0.86 (0.76-0.97) | 0.87 (0.81-0.94) | 0.98 (0.79-1.22) | 0.98 (0.83-1.15) |
| **+ body fat percentage** | 0.86 (0.76-0.97) | 0.87 (0.81-0.94) | 0.98 (0.79-1.22) | 0.98 (0.83-1.15) |
| **+ blood glucose** | 0.88 (0.78-0.99) | 0.87 (0.81-0.94) | 0.97 (0.77-1.19) | 0.98 (0.83-1.15) |
| **+ systolic blood pressure** | 0.86 (0.76-0.97) | 0.87 (0.81-0.94) | 0.99 (0.80-1.23) | 0.98 (0.83-1.15) |
| **+ rice** | 0.86 (0.76-0.97) | 0.87 (0.81-0.93) | 0.98 (0.79-1.21) | 0.97 (0.82-1.14) |
| **+ wheat** | 0.87 (0.77-0.98) | 0.87 (0.81-0.94) | 0.99 (0.80-1.23) | 0.97 (0.82-1.14) |
| **+ poultry** | 0.85 (0.75-0.96) | 0.87 (0.81-0.93) | 1.00 (0.81-1.25) | 0.98 (0.83-1.15) |
| **+ fish** | 0.85 (0.75-0.96) | 0.87 (0.81-0.94) | 1.00 (0.81-1.24) | 0.98 (0.84-1.15) |
| **+ eggs** | 0.87 (0.77-0.98) | 0.88 (0.82-0.95) | 1.00 (0.81-1.25) | 0.98 (0.84-1.16) |
| **+ fresh vegetables** | 0.86 (0.76-0.97) | 0.87 (0.81-0.94) | 0.99 (0.79-1.23) | 0.98 (0.83-1.15) |
| **+ soybean** | 0.86 (0.76-0.98) | 0.87 (0.81-0.94) | 0.98 (0.79-1.22) | 0.97 (0.83-1.14) |
| **+ dairy** | 0.86 (0.76-0.97) | 0.88 (0.82-0.94) | 1.00 (0.82-1.27) | 0.98 (0.83-1.15) |

^1^Analyses were stratified by age-at-risk, sex and region, and were minimally adjusted for education, income, smoking, alcohol intake, BMI, total physical activity, family history of diabetes or cardiovascular disease, and consumption of fresh fruit, red meat and preserved vegetables.


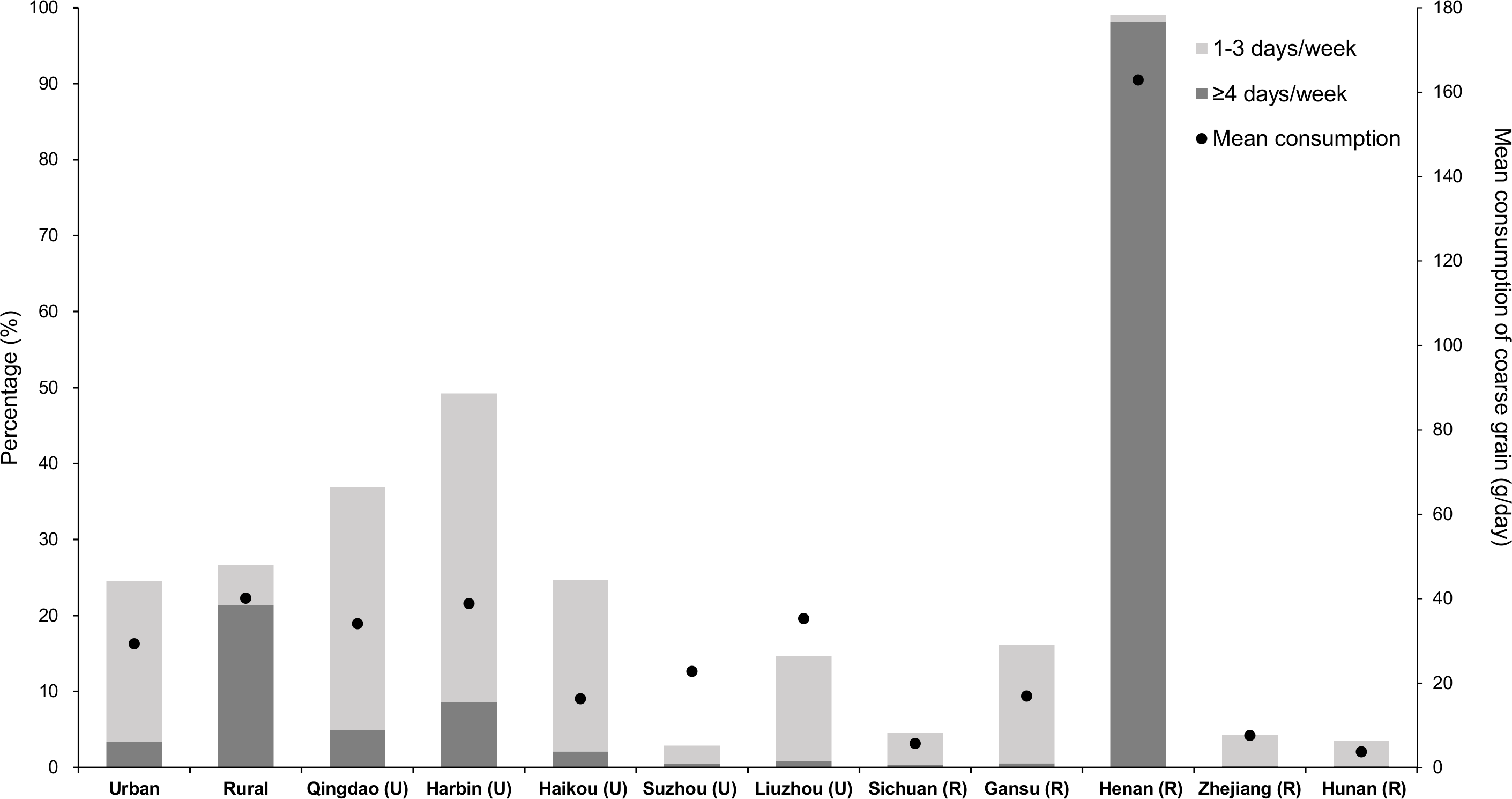


**Supplemental Figure 1.** **Distribution and mean value of coarse grain consumption by region at resurvey in 2013-2014 among Chinese adults.** The bar graph indicates the distribution of coarse grain consumption, with y axis on the left. The point graph indicates the mean consumption of coarse grains, with y axis on the right.


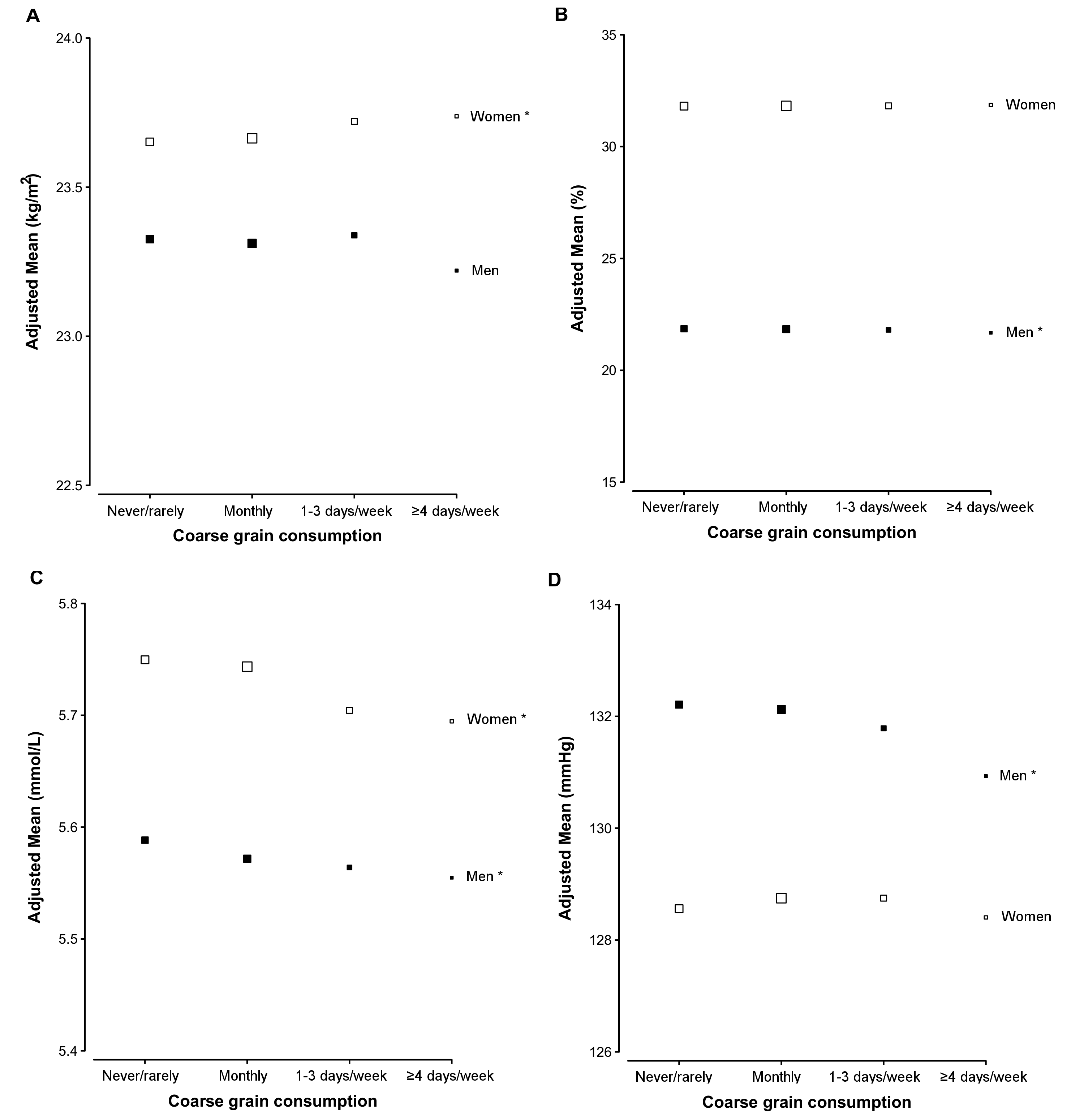


**Supplemental Figure 2.** **Adjusted mean values for A) body mass index (BMI), B) body fat percentage, C) random blood glucose, and D) systolic blood pressure by the frequency of coarse grain consumption among Chinese adults.** Mean values for BMI were adjusted for age, region, education, income, smoking, alcohol intake, total physical activity, and consumption of fresh fruit, red meat and preserved vegetables. Mean values for body fat percentage, random blood glucose and systolic blood pressure were additionally adjusted for BMI. **P* for trend <0.005.


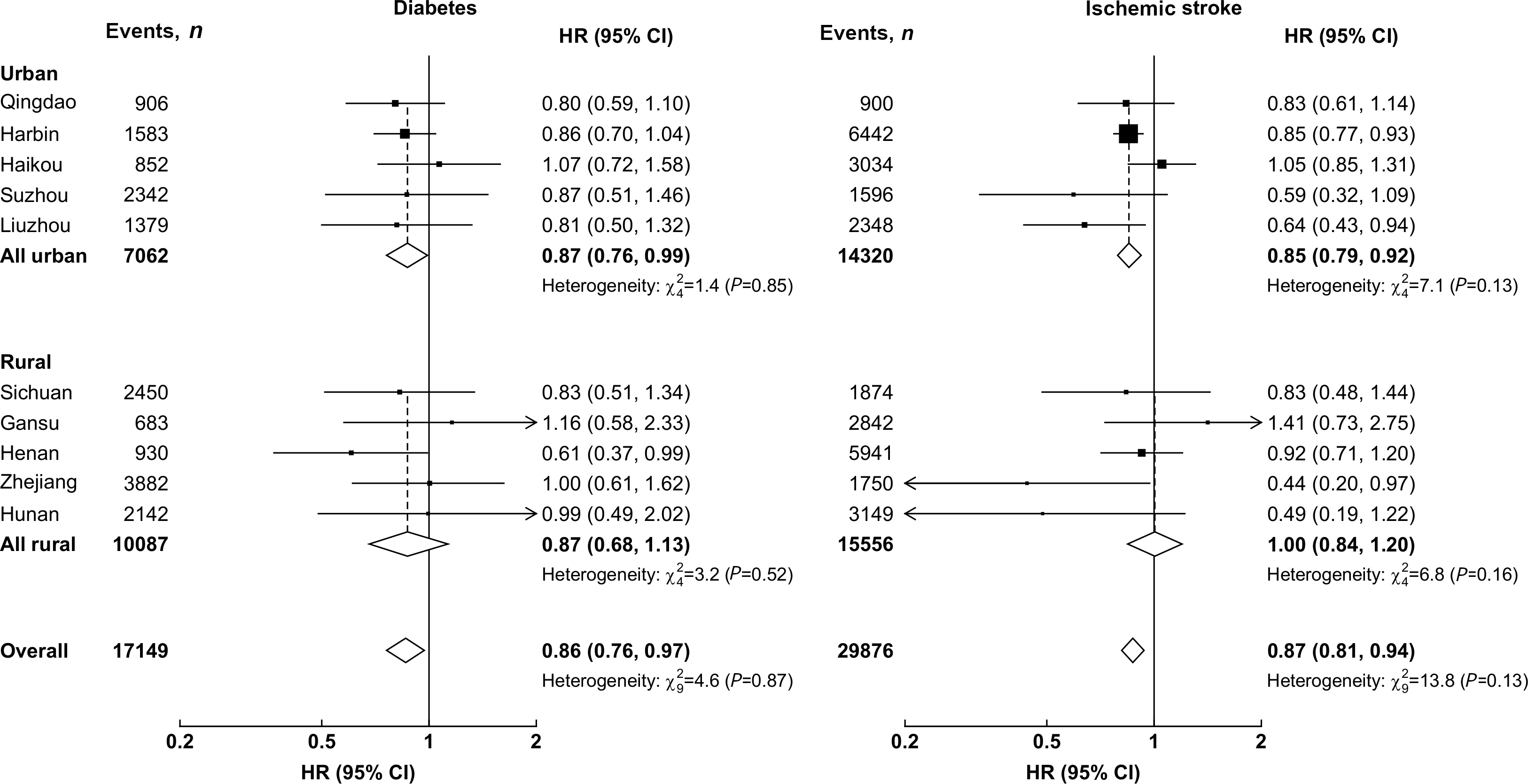


**Supplemental Figure 3. Adjusted HRs (95%CIs) for incident diabetes and ischemic stroke per 100 g/day of coarse grain consumption by region among Chinese adults.** Analyses were stratified by age-at-risk, region and sex, and adjusted for education, income, smoking, alcohol intake, BMI, total physical activity, family history of diabetes or cardiovascular disease, and consumption of fresh fruit, red meat and preserved vegetables. The black boxes represent hazard ratios, with the size inversely proportional to the variance of the logarithm of the hazard ratio, and the horizontal lines represent 95%CI.
